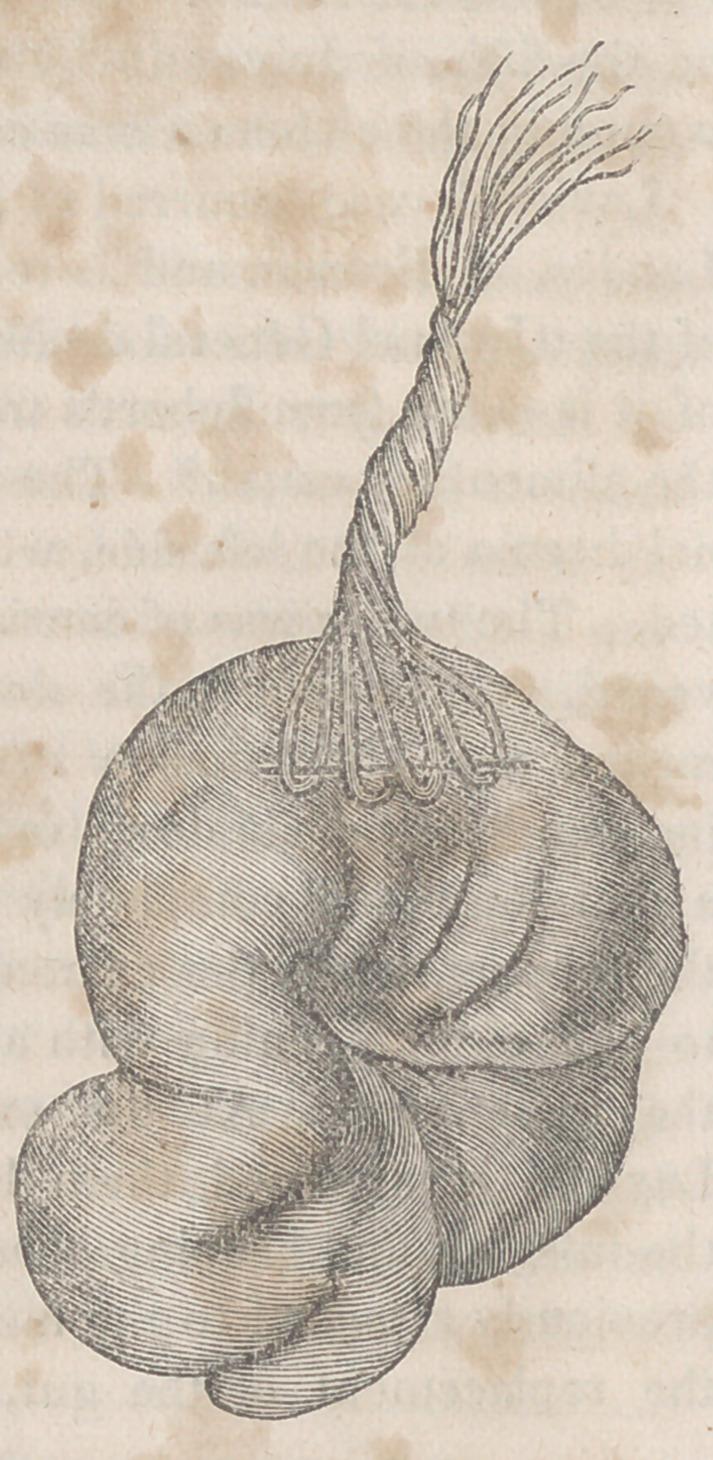# An Experimental and Critical Inquiry into the Nature and Treatment of Wounds of the Intestines

**Published:** 1843-02

**Authors:** Samuel D. Gross

**Affiliations:** Professor of Surgery in the Louisville Medical Institute


### WESTERN JOURNAL

OF

### MEDICINE AND SURGERY.

---

FEBRUARY, 1843.

---

Art. I.—*An Experimental and Critical Inquiry into the Nature and Treatment of Wounds of the Intestines.* By Samuel D. Gross, M. D., Professor of Surgery in the Louisville Medical Institute.

[No. 2.—Continued from page 50.]

Having in the preceding number of the Journal discussed the nature, symptoms, mode of reparation, and therapeutic treatment of wounds of the intestines, I shall now proceed to speak of the different kinds of sutures. In studying this branch of the subject, the reader will be struck with the numerous and diversified expedients that have been devised for the management of this class of injuries.

### I.—*Continued Suture.*

The earliest suture employed for sewing up wounds of the intestines was the glover's, or, as it is generally termed by the French surgeons, the "suture du pelletier." It has also the name of the continued suture, and appears to have been extensively resorted to by the older surgeons in the management of common wounds. It was, however, long ago rejected in the treatment of injuries of this kind, and was for many years entirely abandoned even in cases of enteroraphy of the alimentary canal. Mr. Samuel Cooper, in speaking of this suture, observes that it may, in every point of view, be now considered as totally disused in every case of surgery which can possibly present itself. "When we remember," says he, "in making this suture, how many stitches are unavoidable; how unevenly, and in what a puckered state, the suture drags the edges of the skin together; and what irritation it must produce: we can no longer be surprised at its now being never practised on the living subject. It is commonly employed for sewing up dead bodies; a purpose for which it is well fitted; but for the honor of surgery, and the sake of mankind, it is to be hoped that it will never again be adopted in practice."* How far this sweeping denunciation is entitled to consideration, we shall endeavor to show in another part of this inquiry; it is sufficient, at present, to say that the glover's suture has, in my opinion, been unfortunately too long neglected, and that, when judiciously employed, it is capable of affording the most happy results in the treatment of intestinal wounds, no matter what may be their situation, direction, or extent.

The glover's suture is usually executed with a straight, round needle, armed with a single waxed thread, which is carried from within outwards obliquely from one lip of the

* Dictionary of Surgery, vol. ii, p. 331. New York, 1836.

wound to the other, until the whole track of it is sewed up. The instrument should be introduced throughout at the same distance from the edge of the breach, and the intervals between each two respective stitches must not be too great for fear of fæcal effusion. The thread, moreover, must not be drawn too tightly, otherwise the lips of the wound will overlap each other and have a puckered arrangement; an occurrence which, as it is calculated to interfere with the adhesive process, should be studiously avoided. In performing enteroraphy, the older surgeons were in the habit of leaving at each angle of the wound a length of thread equal to about five inches, which was brought out at the external opening, where it was secured by a strip of adhesive plaster, or by a small compress and bandage. In about six days the ligature was generally sufficiently loose to be withdrawn, or, if it was still pretty tight, the attendant cut it at the middle, and removed it by pulling gently at the ends. During this manœuvre great care was taken to give proper support to the external wound. As executed at the present day, the extremities of the suture are fastened either by a knot, or by passing them under an adjoining loop, after which they are cut off close to the surface of the bowel, into the interior of which the thread employed in the operation ultimately finds its way.

The experiments which I have performed to illustrate the use of the continued suture embrace the subject of transverse.

longitudinal, and oblique wounds, and amount altogether to seventeen in number. I shall detail them in the order in which they are here enumerated.

### a.—*Transverse Wounds.*

Experiment I.—Transverse wound of the arch of the colon two inches in extent—continued suture—recovery.

After considerable difficulty I succeeded in drawing out of the abdomen a portion of the arch of the colon, into which I made a transverse incision two inches in length, and sewed it up with the continued or glover's suture. The dog was large, old, and made much resistance during the operation, which was attended with tolerably copious hemorrhage from the intestinal wound. The stitches were drawn very tight, to insure the more accurate apposition of the divided parts, and considerable violence was done to the omentum and surrounding structures, owing to the unusual restlessness of the animal. Notwithstanding this, he speedily recovered from the shock of the operation, was in excellent spirits the next morning, and had altogether a most rapid convalescence. On the eleventh day after the experiment he escaped from the room in which he was confined, as well, apparently, as if he had not been hurt.

Experiment II.—Transverse section of the ileum—continued suture introduced between the muscular and mucous coats—the animal killed on the twelfth day.

A tarrier was submitted to the same experiment as the preceding, with this difference, that the incision was made into the small bowel, and extended through the entire cylinder. The needle, moreover, was carefully conveyed between the mucous and muscular tunics, instead of through the whole of them, as was the case in the former operation. The wound was fourteen inches from the cæcum. The animal was permitted to live until the end of the twelfth day, when, the cure being considered sufficiently established, he was killed. The small bowel and omentum were extensively glued together by plastic lymph, in a state of organization: the omentum projected into the outer, and adhered to the surface of the inner wound: the suture still retained its hold, though not every where in the same degree, and the villous edges were united by adhesive matter.

### b.—*Longitudinal Wounds.*

Experiment I.—Longitudinal wound one inch long—continued suture—the animal killed at the end of two months and a half.

The subject of this experiment was a large dog several years old; he had fasted for twenty-four hours, and was perfectly well. The wound, one inch in length, was made along the convex surface of the ileum, a short distance from the cæcum, and sewed up with the continued suture with such firmness that nothing could escape through the interstices of the stitches. The day after the operation the animal was well, and continued so, eating and drinking with his accustomed avidity, until he was killed two months and a half after. The outer opening was perfectly healed with a portion of omentum in it. No adhesions existed between the bowels, or between these and the wall of the abdomen. Attached to the outer surface of the intestinal wound was a process of epiploon, which thus served to mark its situation, which it would otherwise have been difficult to detect, so complete was the reparation. The mucous part of the breach was beautifully cicatrized, a slight depression being the only thing out of the way, and the tube retained its normal dimensions throughout. Numerous tape-worms were seen in the small bowel. The various tissues and organs were loaded with fat.

Experiment II.—Longitudinal wound six inches in length—continued suture—the animal killed on the twentieth day.

The dog, as in the last experiment, was large and old. The lower part of the ileum being withdrawn, I made an incision, six inches in length, along its convex surface, sewing up the whole of it with the continued suture. Considerable blood was lost during the operation, which was painful and protracted. The next day the animal was thirsty, looked stupid, and had occasional vomiting. Some blood, which had evidently proceeded from his wounds, was found on the floor of the room. On the third day he was quite cheerful, took some meat that was offered him, and from this time on he rapidly recovered. He was permitted to live until the twentieth day.

The outer wound was perfectly healed, with a small process of omentum intervening between its inner edges. The bowels were free from adhesions, except at the seat of the injury, where two folds were connected to each other and to a piece of the mesentery. On laying open the tube, a vertical fissure, three inches long by three lines in width at the middle, was discovered as the remains of the original wound. The bottom of the chasm was formed by a process of the mesentery, which was firmly attached to the exterior of the bowel, and exhibited a smooth, transparent appearance. The mucous lining was puckered, or thrown into numerous horizontal rugæ, like those of a ruffle, and along the edges of the fissure it was rounded off, elevated, and somewhat irregular. The caliber of the intestine was nearly a quarter of an inch wider than above and below the wound. All the other viscera were healthy, and the animal was in good condition. Every trace of suture had disappeared.

Experiment III.—Wound two inches long—continued suture—the everted mucous membrane pared off—the animal killed at the end of the third month.

The dog, the subject of this experiment, had fasted for twenty-four hours; he was small and several years old. The wound, situated five feet and a half from the ileo-cœcal valve, was two inches long, and united with the continued suture, the stitches being so near each other as to leave no chance for the escape of any thing through their interstices. The everted mucous membrane was carefully pared away, and the whole returned in the usual manner. The recovery was rapid, and three months after the operation the dog was killed. The suture, indeed every trace of it, had disappeared, the breach being thoroughly repaired, and the continuity of the villous membrane re-established. A small process of the omentum adhered to the surface of the affected intestine, and another projected into the outer opening, but was in progress of absorption. The caliber of the tube was in no wise diminished. No adhesions existed between the different convolutions.

Experiment IV.—Longitudinal wound two inches in length—continued suture—the everted mucous membrane pared off—the animal killed at the end of a month.

The dog was small and about eighteen months old; the wound, situated within a short distance of the ileo-cæcal valve, and two inches in length, was closed in the same manner as in the preceding experiment, and the everted mucous membrane pared off on a level with the serous surface. The animal had been fed a few hours previously, and vomited several times immediately after he was removed from the table. The next day he appeared comfortable, and quarrelled with his comrades for his part of the rations. On the 21st of September, precisely one month after the operation, being rather lean, but in good health, he was killed. The omentum, as in the preceding case, adhered to the small bowels, and a process of it was prolonged into the outer wound, which was perfectly healed. There was no appearance of recent peritonitis; a part of the ligature employed in making the suture was still retained, but the wound was beautifully cicatrized, and the cure completely established. The caliber of the tube was natural.

Experiment V.—Longitudinal wound two inches long—continued suture with the everted mucous membrane pared off—the animal killed on the twenty-eighth day.

This experiment, as well as the next two, was merely a repetition of the preceding. The wound, situated four feet from the ileo-cæcal valve, was of the same length and treated precisely in the same manner, the everted villous membrane being cut off close to the peritoneal surface. The animal, small and rather young, was in good condition when he was killed on the twenty-eighth day. The post-mortem appearances did not differ materially from those observed in the preceding case. The outer wound was healed with a piece of omentum in it, and the inner was also nearly repaired, but the suture was only partially detached, being retained by a small slip of mucous membrane. There was no adhesion between the folds of the intestines, or between these and the wall of the abdomen, nor any contraction of the caliber of the tube. In short, the cure was complete.

Experiment VI.—Wound two inches long—continued suture—the everted mucous membrane pared off—the animal killed on the twenty-eighth day.

The subject of this operation was a small young slut; and the wound, not quite two feet from the ileo-cæcal valve, was treated as in the two last experiments. The next day a large piece of omentum, dark, bloody, and covered with dirt, was found protruding from the external wound; it was immediately encircled with a ligature, and excised. The animal, notwithstanding this untoward circumstance, speedily convalesced, and was allowed to live until the twenty-eighth day, when she was killed. The internal wound was nearly healed, but a part of the suture still remained, a few of the stitches not having ulcerated away. The larger part of the thread was lying loose in the bowel, incrusted with solid fæcal matter. The whole would probably have been detached in a few days. The small bowels were slightly united to each other and to the omentum by plastic lymph, and the outer wound was thoroughly cicatrized. The animal had not lost any flesh from the effects of the operation.

Experiment VII.—Wound two inches and a half long—continued suture—the everted mucous membrane pared off—the animal killed on the tenth day.

The dog was large, several years old, and had fasted twenty-four hours. The wound, situated along the convex surface of the ileum, within two feet of the cæcum, was two inches and a half long, and closed as in the preceding experiments, the mucous membrane protruding through the interstices of the stitches being carefully pared away on a level with the serous surface of the bowel. About five ounces of blood were lost during the operation, which was somewhat protracted, owing to the inordinate resistance of the animal. The bleeding had not ceased when the bowel was returned. No untoward circumstance occurring, and the cure being considered established, the dog was killed on the tenth day. A large plug of omentum filled the external wound, the edges of which were already firmly united. The small bowels were extensively adherent to each other and to the epiploon; the suture retained its hold throughout the greater part of its extent, and a layer of lymph occupied the interval between the villous margins of the breach. The tube at the seat of the injury contained fæcal matter, and presented no contraction. The marks of acute peritonitis which generally supervenes upon a lesion of this kind, had entirely disappeared; or, rather, no more inflammation had existed than was necessary to effect the reparation.

Experiment VIII.—Wound one inch long—continued suture introduced between the mucous and muscular tunics—the animal killed on the fifteenth day.

Wishing to ascertain whether the edges of the wound could not be more perfectly approximated by carrying the needle between the muscular and villous tunics, or, in other words, through the cellulo-fibrous lamella, described in a previous part of this essay, I instituted this and the following experiments.

Drawing a loop of the ileum from the abdomen of an old tarrier, I made a longitudinal incision, one inch in length, along its convex surface, not far from the cæcum, and sewed it up by carrying the needle, as just intimated, between the villous and muscular tunics. As had been anticipated, my expectations were not disappointed. The operation, without being more painful or protracted than when executed in the ordinary manner, had the effect of bringing the surfaces of the incision into the most perfect apposition. No severe indisposition followed, and the animal was permitted to live until the fifteenth day, when he was killed and his body carefully inspected. On laying open the bowel, which was closely attached to two adjacent coils, as well as to the omentum, the suture was found to be only partially detached, and to be incrusted with small nodules of fæcal matter. The continuity of the villous surfaces was re-established through the medium of a thin, narrow band of lymph, which was removed by maceration for two days in water. There was no abnormal redness either in the mucous or in the serous coat of the bowel, nor any contraction of its caliber. The continuity of the serous lips of the wound was unusually perfect. The outer opening was healed, a process of omentum being prolonged into it.

Experiment IX.—Wound one inch and a half long—continued suture introduced through the cellulo-fibrous lamella—the animal killed at the end of the thirty-fifth day.

The subject of this experiment, a large dog, several years of age, had fasted for twenty-four hours. The wound, occupying the inferior extremity of the ileum, was eighteen lines in length, and closed precisely as in the preceding experiment. The animal vomited several times within a few minutes after the operation, and appeared considerably exhausted. The next morning, however, he had recovered his wonted activity and cheerfulness, and rapidly convalescing, remained in good health until the thirty-fifth day, when he was killed.

The dissection revealed the following appearences. The outer wound was perfectly healed, and there was no adhesion between the bowels, or between these and the omentum, except immediately around the seat of the injury. No trace of suture was discovered; the villous edges had a rough, granulated aspect, and were united in the greater part of their extent; the wound was scarcely an inch long; the mucous membrane was free from inflammation; and the canal was of the normal dimensions. The dog was rather lean. All the other abdominal viscera were sound.

Experiment X.—Wound three-quarters of an inch long—continued suture introduced through the cellulo-fibrous lamella—the animal killed at the end of thirty hours.

The animal was a small but full grown slut. The wound, situated in the ileum eighteen inches from the ileo-cæcal valve, was nine lines in length, and closed in the same manner as in the last two experiments. The animal did not seem to mind the operation, and was well up to the moment she was killed thirty hours after. The object I had in view in destroying her so soon, was to ascertain the progress which nature had made towards reparation. The outer wound, closed by a plug of omentum, was feebly united by adhesive matter. Three knuckles of the small bowel were agglutinated by plastic lymph, of moderate firmness, with here and there a small ecchymotic speck. The epiploon covered the outer surface of the intestinal wound, and had a red, inflamed appearance for some distance around it. The edges of the villous membrane were of a pale lilac color, flat, and separated only by a very narrow, thread-like band of adhesive matter. There was no contraction of the bowel at the seat of the lesion, and no obstruction to the passage of fæcal matter. A drawing of this specimen was made immediately after it was examined. It was then immersed in dilute alcohol, which had the effect of depriving it in twenty-four hours of its red color, and of detaching the effused lymph.

Experiment XI.—Wound one inch long—continued suture introduced througn the cellulo-fibrous lamella—the animal killed at the end of the fourth day.

Anxious to investigate this point a little further, I repeated the last experiment upon a large dog laboring under an attack of mange. He had fasted for eighteen hours, and bore the operation without a struggle. The wound was twelve lines in length, and situated in the inferior extremity of the ileum, within eleven inches of the cæcum. At the end of the fourth day, without apparently suffering from the effects of the operation, he was killed. The edges of the outer opening were pretty firmly united by adhesive inflammation with an intervening process of omentum. The omentum also adhered to the intestinal wound, as well as for a short distance around it; and the injured part was firmly glued to a neighboring convolution. The lymph which served as the connecting medium was of good firmness, and exhibited all the phenomena of incipient organization. The wound itself was reduced to nearly one-half its original length, and the edges, of a pale rose color, were separated by a thin narrow band of adhesive matter. The villous membrane presented no unnatural redness, nor was there any inflammation of the omentum, except in the immediate vicinity of the injury. No obstruction existed to the passage of the fæces.

Experiment XII.—Wound one inch long—continued suture carried through the cellulo-fibrous lamella—the animal killed at the expiration of forty-eight hours.

The subject of this experiment was a small young slut, four or five months old, which had been fed only a short time before the operation. The incision, an inch long, was made in the lower part of the small bowel, and approximated by the continued suture. She was killed at the expiration of forty-eight hours, having been previously in good spirits. The outer wound was somewhat tumid and but feebly united, a plug of omentum projecting into it. This apron-like membrane had likewise contracted extensive adhesions to the surface of the small intestines, and exhibited all the evidences of high inflammation. The affected cylinder was intimately connected to the adjacent knuckles by plastic lymph, containing a number of small bloody depôts, and readily yielding under the pressure of the finger. On breaking up these adhesions the serous lips of the wound were found to be in close contact with each other, and to be thoroughly coated with the substance just mentioned. The villous edges were of a deep rose color, as was also the mucous surface for some distance above and below, and the ligature retained its situation throughout the whole line of suture; scarcely any lymph intervened between them, and they were perfectly smooth and regular. The bowel was not contracted or diminished in size.

### c.—*Oblique Wounds.*

Experiment I.—Wound one inch long—continued suture introduced through all the tunics, except the serous—the animal killed at the end of the tenth day.

A small, full-grown dog, which had previously fasted, formed the subject of this experiment. The wound occupied the convex surface of the small intestine, three feet from the ileo-cœcal valve, and was closed by the continued suture, the needle being carried through all the tunics, excepting the outer. By this management the serous surfaces were brought into pretty close contact with each other. No untoward symptoms occurring, and the cure being considered established, the dog was killed at the end of the tenth day. The small bowels were extensively connected to each other, as well as to the omentum, and no little difficulty was experienced in finding the wound. The suture still retained its place, except at one extremity of the breach, where it was detached, and hung loose in the canal. The villous edges were somewhat rough and elevated, and intervening between them was a small, narrow band of lymph, interrupted at several points of its extent; the affected part of the tube was of the natural dimensions; the abdominal wound was only partially healed; and a process of epiploon projected into it.

Experiment II.—Wound of the ileum three-quarters of an inch long—continued suture introduced through the cellulo-fibrous lamella—recovery—the dog killed on the twenty-second day.

The wound in this experiment was three-quarters of an inch long, and closed by the continued suture introduced through the substance of the cellulo-fibrous lamella. Its distance from the ileo-cœcal valve was about three feet. The dog, which was young and of middle size, made considerable resistance during the operation, which had the effect of producing some exhaustion, followed by vomiting immediately after he was removed from the table. The next day he was dull and drowsy, but from this time he gradually recovered, and lived until the twenty-second day, when he was killed, being fat and healthy. The small bowels were adherent to each other and to the omentum, but not in so great a degree as in the preceding case. A delicate process of the omentum was attached to the intestinal wound, the villous margins of which were in close contact with each other, their continuity being quite perfect at several points. The suture had ulcerated away, except at the upper angle of the wound, where it still retained a feeble hold. The bowel was of the normal size, and contained semi-fluid fæcal matter. The abdominal opening had healed without the intervention of the epiploon.

Experiment III.—Wound one inch and a half long—suture introduced between the muscular and mucous tunics—recovery—the animal killed on the seventeenth day.

A fold of the small bowel having been drawn from the abdomen of a large dog, twenty hours after he had taken food, an incision, one inch and a half in length, was made along its convex surface, and the edges approximated as in the last experiment. The animal bore the operation without much resistance, and experiencing no ill-effects from it, he was killed on the seventeenth day. The appearance revealed on dissection did not vary materially from those in the preceding cases. The external orifice, only partially cicatrized, had a plug of omentum in it, and this membrane also adhered, though not extensively, to the convolutions of the small intestines. The wounded portion of the tube had contracted very firm adhesions to the mesentery, which thus served to re-establish its continuity. The villous margins, rough and slightly elevated, were in intimate apposition with each other, but the adhesion between them was easily destroyed, except at one point, where the connecting medium was more dense and more completely organized. The breach was not more than thirteen lines in length, unaccompanied, however, with any sensible puckering of the mucous membrane, or diminution of the caliber of the affected cylinder. The suture was loosened in the greater part of its extent, but only partially detached.

The results of these experiments are eminently favorable to the use of the contined suture, as not one proved fatal, although the wounds in several were of extraordinary length.* In eight the needle was carried through the whole thickness of the bowel, and in five, the everted mucous membrane was pared off on a level with the surrounding surface; in eight, the suture was introduced through the fibrous lamella, or between the muscular and mucous coats; and in one, through all the layers of the tube, except the peritoneal. It is worthy of remark that the caliber of the tube was not sensibly diminished by the operation in any of the experiments.

Of these three methods, that of introducing the suture through the cellulo-fibrous lamella is the least objectionable, as it enables us to bring the serous surfaces into more accurate apposition. When the needle is conveyed through all the tunics, there must necessarily be some degree of pucker-

---

*It is proper to state that three of the animals were killed too soon after the operation to render it at all certain that they would have recovered from the effects of it.

ing, whereby the mucous lining will be forced between the lips of the wound, if not beyond the level of the peritoneal membrane. By such an arrangement the adhesive process would be retarded, and if the stitches were to lose their hold, or if the bowel should not become glued to the neighboring parts, fæcal effusion might occur, followed by its whole train of evil consequences.

In making the continued suture I would, therefore, recommend that the needle be carried through the cellulo-fibrous lamella, or between the muscular and mucous membranes, and not across all the tunics, as is generally advised by authors. The lips of the wound should be held parallel with each other during the operation, and the stitches, drawn with considerable firmness, should not be more than a line, or, at farthest, the eighth of an inch apart. The needle is to be introduced a short distance, say half a line, from the peritoneal edge of the opening,* and brought out at the corresponding point on the opposite side. The first stich should be one line from one angle of the wound, and the last about the same distance from the other, care being taken to secure each with a double knot, and to cut off the extremities of the suture close to the surface of the tube. The instrument which I prefer, and which I employed in nearly all my experiments, is a long, slender sewing needle, armed with a waxed and strong but delicate silk thread. The operation should be performed as expeditiously as is consistent with safety, and the bowel handled in the gentlest possible manner.

Mr. Travers, to whose name I have already so frequently referred, and who is one of the most able and influential advocates of the glover's suture, performs the operation with

---

* It should be recollected that in wounds of the bowels there is always considerable retraction of this membrane, by which the other tunics are exposed. Hence if the needle be introduced half a line behind the peritoneal edge of the opening, as recommended in the text, it will be at least the eighth of an inch from the mucous margin, and this will afford the surgeon a sufficient amount of substance to prevent any laceration, or breaking away of the stitches.

a small round sewing needle, armed with a silk thread, and passed near to the lines formed at the bases of the everted lips. The thread is carried at short and regular distances through the whole extent of the wound, the surgeon being mindful that an equal portion of the edges is included in each stitch. When the suture is finished, the ligature is securely fastened, and cut close to the knot. The reduction of the prolapsed fold, he adds, should be conducted with the nicest caution; and he recommends that the outer wound should be treated with a stitch, a plaster, or a poultice, as circumstances may dictate.*

In the management of injuries of this kind, Mr. Travers strongly insists upon the three following points; first, the accurate closure of the intestinal wound; secondly, the careful reduction of the protruded part; and thirdly, the union of the divided integuments. The treatment of the two wounds is thus made perfectly distinct, the internal suture falls into the bowel, and the whole process is materially simplified.

Another advocate of this suture, of no mean authority, was the late Baron Larrey, † whose experience was perhaps more extensive than that of any other surgeon that ever lived. His opportunities for treating wounds of the bowels in the human subject were unusually great, and he likewise performed a considerable number of experiments upon the inferior animals. After having made one or more incisions into the alimentary tube, in different directions, and in dogs of different ages, he united their edges by means of the "suture du pelletier," with the precaution of making it double, using alternately threads of different colors. He directs that the threads should not only be waxed, but anointed with mild cerate, to facilitate their introduction, and that they should be of sufficient length to be left hanging out of the abdominal wound. He advises that they should not be removed before the seventh day, and in some cases not even before

---

* Op. cit., p. 188.

† Surgical Essays, translated by Dr. Revere, p. 233.

the ninth. To extract them, it is only necessary to draw them gently in opposite directions, which may be easily done, as they are of different colors.

Sir Astley Cooper also speaks * favorable of the continued suture; but, like Larrey, he directs the end of the thread to be brought out at the external orifice, which is to be closed with great care. He thinks that cutting off the ligature near the bowel has a tendency to add to the danger of the patient, especially when there is a deficiency in the adhesive process; an opinion for which there is no just ground.

Mons. Velpeau † likewise prefers this suture, of which he has lately suggested the following modification:—In performing it, says he, the needle is carried obliquely down. wards from the upper end of the gut over the outer surface of the lower, from which it is returned to within a line or two of the starting point, passed again to the lower lip, then back to the first, and so on alternately until it has traversed the whole track of the wound. To complete the operation nothing more is necessary than to draw in opposite directions the ends of the ligature, one of which will be at the origin, the other at termination of the suture. The object of this traction is to invert the edges of the wound and bring the serous surfaces into contact, but as this does not always answer, it may be proper to use a probe or catheter. The operation is finished by making a double knot. In mortified hernia, the ends of the thread, or even one of them, would be sufficient for retaining the bowel behind the ring, supposing it was not desirable to let it slip into the belly; and in this event the knot would also be unnecessary.

I shall conclude this subject with the following cases, which, so far as I know, are the only ones in which the continued suture was employed in the human subject, or, rather, in which the particulars have been communicated to the pro-

* Lectures, by Tyrrell, p. 497.

† Medicine Operatoire, T. 4, p. 138.

fession. From the antiquity of the operation, however, there can be no doubt that it has been often resorted to by practitioners, and it is to be regretted that our information respecting it is so limited.

Case I.—**Two perforations of the ileum with a knife—protrusion of the bowel —each opening closed with a continued suture—recovery in five weeks.***

Antonia Josie da Costa was stabbed, on the 3d of August, with a knife in the right hypogastric region, about three fingers' breadth above the pubic bone, the wound in the peritoneum being about nine lines in length. Through this opening a portion of the ileum protruded about ten or twelve inches, and presented two apertures opposite each other large enough to admit a finger. After clearing away the grumous blood, Mr. Peter Travers, a surgeon of Lisbon, who attended the case, closed each perforation with an uninterrupted suture, the ends of which were brought out at the external wound, which was sewed up in the usual manner. During the first four or five days after the accident, the man had severe pain in the abdomen, high fever, frequent vomiting, and hiccough. By repeated bleedings and clysters these symptoms gradually subsided, and towards the end of the fifth day he had a natural alvine evacuation, The internal sutures came away spontaneously on the twelfth of August, and on the seventh of September the patient was discharged in good health, the outer wound being entirely cicatrized.

Case II.—**Wound of the colon attended with the escape fæces—patient eighteen years of age—continued suture—recovery.**

This case is reported by Glandorpius,† an old surgeon, but it is deficient in some important details, a circumstance which detracts considerably from its value. The patient was a young

* The Philosophical Transactions of the Royal Society of London, abridged, vol. xi., p. 73.

† Speculum Chirurg. Obs., 34— Travers, op. cit., p. 168.

man 18 years of age, and the wound, the size of which is not stated, occupied the colon, and permitted the fæces to escape externally. Glandorpius employed the glover's suture, and although the symptoms were for sometime of a very unpromising character, complete recovery ensued. In another case, in which the wound implicated the ileum, and was plentifully besprinkled with an astringent powder, the patient died of gangrene on the fourth day.

Case III.—Two transverse wounds of the small bowel—continued suture—attachment of the mesentery to the outer opening by two ligatures—recovery in thirty-six days.*

An Austrian soldier, in a scuffle with one of his comrades, was stabbed with a knife in the right side of the abdomen, about an inch above the umbilicus. The wound was transverse, about three inches long, and gave vent to a very considerable quantity of the small intestines. The patient being immediately conveyed to the Hotel-Dieu at Châlons-sur-Marne, Mons. Charliar, the surgeon-in-chief, discovered that the protruded gut was divided in two places; at one, in about one-half of its circumference, and at the other, about one-fifth. Passing a loop of thread through the mesentery behind each injured knuckle, he sewed up the wounds with the continued suture, and returned the whole into the peritoneal cavity. The two sutures were maintained near the edges of the outer opening, by means of the threads in the mesentery, which were fastened by an appropriate bandage to the surface of the abdomen. Emollient fomentations were applied to the belly, and the patient was kept in the semi-erect posture by pillows placed behind his back. For a month the most rigid regimen was observed. The ligatures were withdrawn at the end of a few days, as soon as it was was found that the intestine had contracted firm adhesions to the inner surface of the wall of the abdomen. The outer

* Dictionnaire des Sciences Medicales, T. 43, p. 48.

wound cicatrized rapidly, and the patient left the hospital perfectly cured on the thirty-sixth day.

Case IV.—Large sabre wound of the ileum—extensive protrusion of the small bowel—escape of stercoraceous matter—patient twenty-three years of age—continued suture—complete recovery in less than seventy days.*

The subject of this case was John Baptist Jolin, about twenty-three years of age, a fusileer in the sixteenth regiment of the guard. While playing with one of his comrades, he fell by accident upon the point of his sabre, which he held unsheathed in his hands, and which made a deep wound in the abdomen. He was carried to the neighboring village of Pucteau, where Mons. Carré sewed up his wounds. The external opening, about fifteen lines in extent, occupied the lower part of the right side of the abdomen, and gave vent to a large portion of the ileum which was already tumefied. "I examined the protruded bowel," says Mons. Carré, "and found a large wound, attended with a discharge of stercoraceous matter, which obliged me to make a suture at this point, immediately after which I returned the intestine into the cavity of the abdomen, dressed the parts, and sent the patient to the Hospital of the Guards at Paris." During the journey, which was tedious, he vomited copiously, and had one bloody stool.

On removing the dressings, immediately after his arrival in Paris, a portion of the small intestine, which had become prolapsed during the journey, and presented a swollen appear-

---

* I am indebted for this and the following case to Baron Larrey. The first is recorded in his Surgical Essays, edited by Doctor Revere; the other in his Memoirs of Military Surgery and Campaigns of the French Armies, translated by Dr. Hall of Baltimore, (vol. ii. p. 387.) Although it is not positively stated that the suture employed in these cases was the continued, yet there is strong reason to believe that it was, both from the size of the wound, the nature of the operation, and the decided preference which he has expressed for this method in different parts of his writings.

ance, was returned into the cavity of the abdomen without much effort. The patient, however, was not relieved. He was extremely weak and anxious, and had frequent vomiting of bilious matter, accompanied with violent colicky pains, tenesmus, and small bloody stools. On visiting him in the morning, Baron Larrey unbound the wound of the integuments and the opening made by the sabre in the aponeurosis of the great oblique muscle, when he discovered that a considerable quantity of blood had been effused into the peritoneal cavity, and that several of the convolutions of the intestines had already become united to each other. He therefore contented himself, although the symptoms of strangulation still remained, with evacuating the extravasated fluid, and dressing the wound with a linen rag, spread with styrax ointment, the whole being secured by a suitable bandage.

For thirteen days the symptoms were of the most violent character, and the patient was only saved by repeated dry and moist cupping of the abdomen, followed by camphorated embrocations and anodyne cataplasms, and finally by the application of blisters, with the use of enemeta and the most rigid abstinence. At this period a small ligature, about three inches and a half long, was discharged through the external wound, and there was immediately a striking amelioration of all the symptoms. The patient grew better and better; the wound of the abdomen soon cicatrized; and in less than seventy days from the accident he was completely cured.

Case V.—Two sabre wounds in the colon—extensive division of the mesentery—hemorrhage into the peritoneal sac—continued suture with the ends brought out at the the abdominal opening—death on the seventh day from inflammation and gangrene of the peritoneum and intestines.

A grenadier was wounded with a sabre in a duel, on the right side of the umbilical region. A considerable portion of the small bowel protruded across the opening, and presented a reddish brown appearance; it was inflated, and contained a collection of worms. The patient suffered much pain and distressing anxiety; the pulse was small and thready; the countenance ghastly; and the extremities cold. In this state he had been seven hours when he was brought to the hospital. Baron Larrey immediately dilated the abdominal opening, to relieve the strangulation, and to examine the other portions of the tube to see whether it was injured. He found the small curve of the colon wounded in two places, and the mesentery extensively divided by the sword. Having extracted the worms, which were still alive, with a pair of dressing forceps, he introduced a suture through the lips of the wounded intestine, and after bathing it with warm wine, reduced it, taking care to retain the ends of the thread on the outside. A considerable quantity of black clotted blood escaped at this stage of the operation, showing that effusion of this fluid had taken place in the abdomen. The lips of the external wound were approximated by a compress and roller. The patient was conveyed to bed, and took two grains of opium in sweet wine, which allayed his suffering and promoted reaction. The next day the abdomen was painful and tender to the touch; the urine was suppressed; the skin hot, and the thirst urgent. The edges of the outer wound had separated, but presented nothing remarkable. He died on the seventh day from inflammation and gangrene of the peritoneum and intestines.

On dissection the portion of bowel, formerly protruded, was found nearly of the natural appearance. The edges of the inner wound were agglutinated to each other, while those of the mesentery lay in folds, being united by adhesive substance, so that it was impossible for any alvine matter to escape into the peritoneal sac. The pelvis and interstices of the viscera were occupied by black and decomposed blood. Extensive adhesion existed among the different organs; the lower part of the ileum was sphacelated at several points; and the superior mesenteric artery was divided near its origin. But for the latter injury, it is nighly probable, as has been remarked by Baron Larrey, that the man might have survived the wounds in the intestines, and finally recovered.

### II.—*Interrupted Suture.*

It is not easy to determine, at this remote period, when, or by whom, the interrupted suture was first introduced to the notice of the surgeon. There can be no doubt, however, that it has been in use almost from time immemorial. The manner of performing it is too well known to require any mention in this place. The following experiments and observations will sufficiently illustrate the value of this suture in the treatment of wounds of the intestines. The former are arranged, in reference to their direction, into three classes, namely, into transverse, longitudinal, and oblique.

#### a.—*Transverse Wounds.*

Experiment I.—Complete section of the small bowel—four interrupted sutures—the ends of the threads cut off at the knots—death from peritoneal inflammation in forty-five hours.

Having opened the abdomen of a large dog after he had fasted for twenty-four hours, I drew out a fold of the small bowel by means of a blunt-hook, and divided it as far as the mesentery. The edges of the wound were then brought together by four interrupted sutures, placed equidistant from each other, and the ends cut off close to the serous surface. The whole being returned into its natural situation, the outer opening was united by two stitches, and the animal allowed water but no food. The operation was performed at eleven o'clock in the morning. In the afternoon the dog was sick at the stomach, threw up water several times, and lay quietly on his side. His thirst was not urgent, nor did he seem to suffer much pain. The next day he was dull and heavy, with occasional vomiting; his breathing became short and laborious, and he died in a state of coma forty-five hours after the experiment.

The following appearances were observed on dissection.

A process of omentum, very red and slightly adherent to the surrounding parts, projected into the outer opening, the edges of which were united by lymph. The peritoneal sac contained a pint of sanguinous fluid, and was universally inflamed. Three knuckles of the small bowel adhered to each other, and the wound was every where covered with plastic lymph, except at one point, three lines long, where the closure was imperfect, and where there had evidently been an escape of alvine fluid. The mucous membrane at the wound was slightly everted and rounded off, and exhibited all the evidences of high inflammation. The bowel above the seat of the injury was obstructed with fæcal matter, of a solid nature, produced apparently by a palsied state of the muscular fibres, and not by any contraction of the canal.

Experiment II.—Section of the entire cylinder of the bowel—seven interrupted sutures—recovery—the animal killed on the seventeenth day.

A small slut was submitted, Semptember 4th, to the same experiment as the preceding, with this difference, that the wound was closed with seven interrupted sutures instead of four, about two lines from each other. She bore the operation well, and lived without any untoward symptoms until the seventeenth day, when she was killed. The external wound was beautifully healed, and a considerable quantity of adeps was found beneath the skin of the abdomen. There was no adhesion of the bowel or omentum to the parietal portion of the peritoneum, and the internal wound, situated within a few inches of the ileo-cœcal valve, was in great measure healed; three of the sutures, however, still retained their hold. No evidence of inflammation was discoverable at the seat of the injury, and the tube had undergone no contraction.

Experiment III.—Transverse wound embracing three-fifths of the cylinder of the small bowel—three interrupted sutures—the ends of the ligature cut off near the knots—recovery—the animal killed near the end of the third month.

May 23, I divided the small intestine eighteen inches from the ileo-cœcal valve three-fifths across, making a wound about fourteen lines in extent, the edges of which were brought together by three interrupted sutures, at equal intervals from each other, and the ends cut off as in the preceding experiment. The animal, which was healthy and of moderate size, had been fasting for twenty-four hours. No blood was lost during the operation, from the effects of which he speedily recovered. On the 16th of August, being in good condition, he was killed, and the body carefully inspected. The outer wound was perfectly cicatrized, as was also that in the bowel. The latter, however, was somewhat rough on its external surface, from the attachment of a small narrow process of the epiploon, which was partially ulcerated, and would doubtless in a few days more have lost its entire connexion. Internally the reparation was beautifully perfect. No adhesion existed between the bowel and the wall of the abdomen, or between any of the intestinal convolutions.

Experiment IV.—Transverse wound occupying four-fifths of the circumference of the tube—four interrupted sutures—the dog killed on the ninth day.

In this experiment, which was performed on a middle-sized dog after he had fasted for eighteen hours, the small bowel was cut four-fifths across, three feet and a half from the ileo-cœcal valve, and the wound united by four interrupted sutures, equidistant from each other. One end of each ligature was brought out at the external opening, and the other cut off close to the knot. The animal rapidly recovered from the shock of the operation, and lived until the ninth day, when he was killed. The protruding ligatures were detached, and the edges of the external opening firmly united, with a small process of omentum intervening between them. The internal wound was partially cicatrized, and exhibited well marked traces of the situation of the sutures. The parts immediately around were rough and knobby, from the presence of lymph and adherent omentum; but there was no abnormal vascularity or evidence whatever of inflammatory action. The bowel contained fæcal matter, and was as large as natural. The animal had not lost any flesh.

Experiment V.—Transverse section embracing five-sixths of the intestinal cylinder—four interrupted sutures with both ends cut off—the animal killed in three weeks.

The subject of this experiment was a small pup not more than five or six months old. The small bowel was cut five-sixth across, and the everted mucous membrane pared away on a level with the peritoneum. I then passed the needle through all the coats of the intestine, hoping thereby to approximate more completely the serous surfaces. In this, however, I found myself mistaken; for no sooner did the instrument enter the muscular lamella than violent contractions ensued, producing fully as much eversion as before. Four interrupted sutures, with the ends cut off close to the peritoneal surface, were employed, and the whole returned within the abdomen. The dog became sick a short time after the operation, and had repeated vomiting; but he gradually recovered, and escaped, at the expiration of the third week, in good health.

Experiment VI.—Semi-division of the bowel—two interrupted sutures with one end protruding—the animal killed on the thirty-sixth day.

On the 10th of August, I opened the abdomen of a tarrier slut, and cut the small bowel half across, three feet from the ileo-cœcal valve, sewing up the wound, which was about three-quarters of an inch in length, with two interrupted sutures, one end of which was left hanging out at the external opening. The animal experienced apparently very little inconvenience from the operation, and was killed in good health on the 16th of September, or the thirty-sixth day after. The external wound had healed without the intervention of the omentum, which, however, adhered to several intestinal folds. The internal wound was beautifully cicatrized; all trace of suture had disappeared, and there was no mark whatever of recent inflammation, either in the serous or mucous tissues. The tube was of the normal size.

### b.—*Longitudinal Wounds.*

Experimext I.—Longitudinal wound two inches long—four interrupted sutures—the ends of the ligatures cut off near the peritoneal surface—the animal killed after the third month.

An incision, two inches in length, being made along the convex surface of the ileum, a little more than a foot from the ileo-cœcal valve, I approximated the edges with four interrupted sutures, equidistant from each other, and cut off the ends close to the peritoneal surface. The dog, which was large and old, lost a good deal of blood during the operation, and the wound was still bleeding, though not freely, when I returned the bowel into the abdomen. He rapidly recovered from the effects of the injury, and continned in excellent health until he was killed on the 22d of September, upwards of three months after the experiment. The epiploon adhered to the surface of the bowel, and a small process projected into the outer wound. The interval wound was perfectly cicatrized, so much so, that some difficulty was experienced in determining its situation: two coils of the intestine were united together at the seat of the injury. The dog was fat, and all the viscera were free from disease.

Experiment II.—Longitudinal wound one inch long—four interrupted sutures—the ends of the ligatures brought out at the abdominal opening—the animal killed on the twelfth day.

A large healthy dog, having fasted for twenty-four hours, was subjected to the same experiment as the preceding, with this difference, that the wound was only one inch long, and that the ends of the ligatures were brought out at the external opening. Nothing unusal occurred after the operation, which he bore with comparatively little resistance. On the twelfth day, being considered out of danger, he was killed. The abdominal wound was cicatrized throughout its entire extent with the intervention of a plug of omentum, a small mass of which also adhered to the injured bowel. The latter was slightly agglutinated to several of the neighboring coils, and on laying it open the villous portion of the breach was found to be well repaired, the edges being rounded off, and connected by plastic lymph. The tube at the seat of the lesion contained alvine matter, and was of the natural diameter. No inflammation was observable either in the serous or mucous coat. It should have been stated that all the ligatures were detached on the sixth day.

Experiment III.—Longitudinal wound one inch in length—four interrupted sutures—the ends of the ligatures cut off close to the knots—the animal killed on the twenty-second day.

This experiment was merely a repetition of the last. Instead, however, of bringing the ends of the ligatures out at the abdominal wound, they were cut off close to the peritoneal surface. The dog was small but full-grown, and had fasted for twenty-four hours. He bore the operation remarkably well, appeared very sprightly soon after it was over, and drank a considerable quantity of water. No untoward symptom arose, and he continued perfectly well until the twenty-second day, when he was killed. The appearances on dissection were found to be essentially the same as in the cases already mentioned. A piece of omentum was attached to the entire surface of the intestinal wound, the reparation of which was unusually perfect. All trace of suture had disappeared, and the continuity of the villous surface was, in a great degree, re-established. There was some adhesion between the neighboring folds of the small bowel, and a process of the epiploon was prolonged, as usual, into the abdominal wound.

Experiment IV.—Longitudinal wound five-eighths of an inch long—two sutures with the ends brought out at the external wound—recovery.

The subject of this experiment was a small dog four or five years old. The wound, five-eighths of an inch in length, was made, as usual, along the convex surface of the bowel, and closed with two interrupted sutures. The ends of the ligature were twisted together, and left hanging out at the external opening. The animal bore the operation well, and was apparently in perfect health a few hours before his escape, which happened at the end of the ninth day.

Experiment V.—Longitudinal wound half an inch long—one suture with the ends cut off at the knot—the animal killed at the expiration of the seventh day.

From the abdomen of a large slut I drew a fold of the ileum within fifteen inches of the ileo-cœcal valve, and making a longitudinal incision along its convex surface, six lines in length, I closed it with a single suture, the ends of which were cut off at the knot. No untoward symptom arising, and being considered past all danger from the operation, she was killed at the expiration of the seventh day. The outer opening was pretty well healed, with a portion of epiploon adhering around its inner margins, as well as to the surface of the intestinal wound, and to several neighboring knuckles. The suture had disappeared, reparation was going on beautifully between the villous edges, and the bowel, containing fæcal matter, had experienced no change in its caliber. The quantity of lymph poured out by the peritoneal surface was very small, as was proved by the trifling nature of the adhesions. The dog was fat, and had lost no flesh from the operation.

Experiment VI.—Longitudinal wound half an inch in length—one suture with the ends cut off close to the knot—death on the eleventh day from causes apparently unconnected with the operation.

This experiment was merely a repetition of the last. Although only one suture was employed, it seemed to have the effect of closing the opening completely. The dog, which was very small and not more than three or four months old, bore the operation exceedingly well, and had no untoward symptoms prior to his escape, which took place three days thereafter. He remained at large until the eleventh day, when he was met in the immediate vicinity of the dog-house in a paroxysm of convulsions, in which he was knocked on the head and killed. On dissection I noticed the following appearances. The outer opening was perfectly healed, except at one or two points, with a small slip of omentum prolonged into it. No peritoneal inflammation was observable any where; the bowels did not adhere to each other or to the abdominal walls; the ligature was detached; and the continuity of the affected portion of the tube was re-established by a process of the epiploon, which was firmly attached around the wound for some distance. The edges of the latter were one-third of an inch apart at the centre; the mucous membrane was not at all red or inflamed; and the small intestine was empty and blanched. What caused his convulsions I could not determine.

### c.—*Oblique Wounds.*

Only two experiments were performed with a view of illustrating the treatment of oblique wounds by the interrupted suture, and of these the following synopsis must suffice.

Experiment I.—Wound six lines long—two interrupted sutures through the cellulo-fibrous lamella—the animal killed at the end of the third week.

The dog, old and of large size, had fasted for nearly twenty-four hours. The wound was made along the convex surface of the small bowel, opposite the attachment of the mesentery, and about twenty inches from the ileo-cœcal valve: it was six lines in length, and closed with two interrupted sutures carried through the cellulo-fibrous lamella. The ends were cut off near the peritoneal surface. Nothing worthy of notice occurred after the operation, and the dog, being in good health, was killed at the expiration of three weeks. On opening him I found that both sutures had disappeared, leaving merely a slight circular depression which served to indicate their former situation. The mucous portion of the wound presented a linear or fissured aspect, and might be said to be in great measure cicatrized. The serous surface had contracted firm adhesions to the mesentery, and the tube retained its normal dimensions. The omentum adhered slightly to the small intestines, and a process of it was prolonged into the outer wound, which was completely consolidated.

Experiment II.—Wound one inch and a half in length—five interrupted sutures—the ends of the ligatures cut off close to the knots—the animal killed upwards of a month after the experiment.

The animal was small, but full-grown, and the wound, eighteen lines in length, was united by five interrupted sutures, equidistant from each other. The ends were cut off close to the knots. The wound, as in the foregoing experiment, occupied the inferior extremity of the ileum. The dog soon recovered from the shock of the operation, and was killed one month and three days after. The sutures had all disappeared, and the wound was neatly cicatrized throughout, excepting at two or three small points, where the edges were somewhat elevated and puckered. It had diminished nearly six lines in length. The tube was slightly contracted at the seat of the injury, but not sufficiently to interfere with the transmission of the alvine matter. Externally the bowel adhered firmly to two adjacent convolutions, together with a fold of the epiploon. The abdominal wound was entirely healed.

Of the foregoing experiments, two were fatal, death being produced in one by peritoneal inflammation, in the other without any obvious cause. The suture was carried through all the tunics in thirteeen, and in one through the cellulo-fibrous lamella. In ten, both ends of the threads were cut off close to the peritoneal surface; in two, they were brought out at the external opening; and in the remainder, one extremity was cut off, and the other secured to the surface of the abdomen.

The most important circumstances to be observed in making this suture in wounds of the bowels are, to carry the needle through the cellulo-fibrous lamella, and to place the stitches sufficiently near each other to prevent the escape of fæcal and other matters. It will be perceived that in some of the above experiments the interval between the stitches was as much as four or five lines, and yet no effusion occurred. Such a practice, however well it may answer in the inferior animals, is certainly inapplicable to the human subject, whose safety should never be jeoparded by inattention to the dictates of sound experience.

It will be seen that in these experiments, as in those with the continued suture, the caliber of the intestinal tube was not seriously diminished in a single instance. Indeed, in nearly all the cases in whieh the parts were examined, it was quite as large at the seat of the injury as in the natural state·

I subjoin the following cases in which the interrupted suture was employed in the human subject. Taken in connexion with the experiments just detailed, they exhibit an array of success highly favorable to this method of treatment.

Case I.—Extensive wonnd of the abdomen, with complete division of the ileum, and penetration of the thoracic cavity—four interrupted sutures—recovery in a month.*

Henry Cooper, seven years of age, had his belly ripped open by a boar on the 23d of August, 1815, soon after eating a small piece of bread and bacon with two apricots.

* Edinburgh Medical and Surgical Journal, vol. xii, p. 27.

Nearly the whole of the abdominal viscera, the stomach, a large portion of the intestinal canal, the mesentery, and omentum were protruded through an immense wound on the left side of the median line. The ileum was completely severed, the omentum was lacerated through its entire extent, and a rent an inch long existed in the mesentery. On the left side of the chest was a lacerated wound five inches long, communicating with the cavity of the chest, and complicated with fracture of the fifth and sixth ribs. The wound in the wall of the abdomen commenced about an inch below the antero-superior spinous process of the iliac bone, from which it reached, in an oblique direction, to the right side of the ensiform cartilage of the sternum. Mr. Calton, surgeon at Collingham, brought the edges of the intestinal wound together by four interrupted sutures, made with a small curved needle and a double silk thread. A firm hold was taken by carrying the instrument through all the tunics of the gut, and each ligature was cut off close to its knot. The omentum was returned without any other attempt to approximate its divided edges than laying them together. The wound of the abdomen was secured by sutures and adhesive straps, supported by a broad bandage.

For the first forty-eight hours after the occurrence of the accident there was great restlessness, high fever, tenderness and tumefaction of the abdomen, and irritability of the stomach. On the twenty-fifth the boy vomited up some undigested ham, two apricot-stones, and several lumbricoid worms, with great mitigation of his symptoms. The following day he had a copious liquid evacuation from his bowels, from the administration of a dose of castor-oil, aided by an enema of sulphate of magnesia. The external sutures came away on the twenty-ninth and thirtieth of August, and in a month the wounds were perfectly cicatrized. The little patient was of course much emaciated, and the wall of the abdomen, at the original seat of the injury, was so thin as to allow the peristaltic action of the intestine to be plainly seen through it.

Case II.—Four wounds of the small bowel with injury of the omentum—treatment by the interrupted and continued suture—recovery in a fortnight.*

An athletic young man, twenty years of age, in carelessly handling a scythe, on the 26th of August, 1836, with the point turned towards the body, accidently pierced his belly a little above the left of the umbilicus, inflicting a longitudinal wound five inches in length externally, but not quite so extensive within. The omentum was perforated and the jejunum opened in four places. One of the wounds in the latter reached nearly entirely across the gut, the second rather more than one-half, the direction of both being transverse: of the other two, one was a mere puncture, yet sufficient to allow the escape of fœcal matter, while the other, which was a longitudinal slit, was two inches in length. A large mass of the small intestines, a portion of the colon, and the omentum protruded through the outer wound, and were found, nearly four hours after the accident, covered with dried blood and fæces, the latter of which had issued in considerable quantities. The patient was in great suffering, his symptoms resembling those of strangulation of the bowels, which, indeed, was the case. After thoroughly cleansing the protruded viscera with tepid milk and water, Dr. Aquila Toland, of Madison county, Ohio, who attended the patient, brought the lips of three of the wounds together by the interrupted suture, made with broad linen threads. The large transverse cut was treated with the glover's suture, that is to say, the two small needles which he used for that purpose were passed alternately from one side to the other between the mucous and muscular tunics, the former being pushed back and excluded from the ligature. The parts were then returned into the abdomen, having previously somewhat enlarged the outer

---

* Western Journal of the Medical and Physical Sciences, vol. 10, p. 481.

wound, the edges of which were next brought into apposition and retained by the quilled suture. Two days after the operation a small process of omentum was discovered in the lower angle of the external opening, upon pushing back which, a copious discharge of bloody pus occurred, followed by great relief of the hypogastric distention. On the thirty-first of August the patient had, for the first time since the accident, a free evacuation from the bowels; on the fourth of September, all unfavorable symptoms had disappeared; and in a fortnight the outer wound was nearly cicatrized.

Case III.—Perforation of the small bowel by the horn of an ox—interrupted suture—dilatation of the abdominal wound—complete recovery.*

William Kemble, twenty-one years of age, of spare habits, a butcher by occupation, was gored in attempting to slaughter an ox, December 11th, 1832, the horn penetrating the abdomen just above Poupart's ligament. Through this opening about six inches of small intestine protruded, which was at the same time strangulated. On examining the gut it was found to have been completely perforated by the animal's horn, which had entered it on one side and come out at the other, producing consequently two apertures, capable each of admitting a finger. No fæces had escaped, nor had there apparently been much hemorrhage. Mr. J. D. Davids, a surgeon of Cowes, being called to the case, immediately brought the lips of the larger wound together with two sutures, and those of the smaller with one, the ends of the ligatures being cut off close to the knots. He then attempted to return the bowel, but found this impracticable without dilating the external wound, which was accordingly done with a probe-pointed bistoury. The outer opening was closed with sutures, supported by straps of adhesive plaster. For the first few days there was considerable restlessness, with vomiting and tenderness of the abdomen. On the fifteenth

*Medico-Chir. Review, vol. xx, p. 182.

some oil was given by the mouth, which acted very well on the bowels, and from this period he went on progressively improving. One of the sutures only made its way out through the wall of the abdomen, the two others fell into the intestinal canal, and were passed with the fæces. Complete recovery ensued.

Case IV.—**Oblique wound of the small intestine, three-fourths of an inch long—four interrupted sutures with the ends cut off close to the peritoneal surface—recovery in five weeks.***

M. Sullivan, aged twenty-six years, a native of Ireland, was admitted into the New-York Hospital, under the care of Dr. Buck, on the 17th of August, 1840, with a stab in the abdomen, received an hour before in a quarrel. The external wound, which was situated on the left of the median line, midway between the pubes and umbilicus, was an inch long, and gave vent to several knuckles of small intestine, in one of which was an oblique cut three-fourths of an inch in extent. The protruded parts were of the natural warmth and of a deep red color; the patient was faint and restless; he had frequent vomiting, with insatiable thirst; and the pulse was weak and small. Four sutures of fine silk thread were introduced into the inner wound; they included all the tunics of the bowel, and the ends were cut off close to the knots. Reduction was then attempted, but did not succeed until the outer cut was dilated to the extent of half an inch at its upper edge. The outer wound was then united with two sutures and adhesive strips, the whole being supported by a broad bandage. During the first day ten ounces of blood were taken from the arm, and four dozens of leeches applied to the abdomen. On the 18th the patient was comfortable; there was pain, however, on pressure at the seat of injury; and towards evening, the pulse having increased in force and tension, the venesection was repeated to

* New-York Journal of Medicine and Surgery, No. 8, April, 1841.

twenty ounces. On the 19th he made several fruitless attempts at stool, and the belly became tympanitic and somewhat swollen; for these symptoms a large blister was applied, and an emollient enema administered. On the 23d of August he was leeched on the right iliac region, and from this period his convalescence was completely established; the bowels moving spontaneously or by the aid of injections, and the tenderness disappearing from the abdomen. The external wound healed kindly, partly by the first intention; and in about five weeks he began to sit up. His recovery, however, was retarded by an attack of inflammation of the chest, and effusion into the cavity of the peritoneum. These gradually yielded to appropriate treatment, and he left the hospital on the 28th of October.

Case V.—Two incised wounds of the small intestines, each more than half an inch long—interrupted suture—recovery in a fortnight.*

Ezekiel, an athletic negro, aged thirty years, in a night broil, was wounded in the arm and abdomen with a knife, the latter injury involving one of the small bowels, which was cut in two places to the extent of more than half an inch. Several branches of the mesenteric artery were divided and bled freely. The bowel protruded through the wound. Having washed off the coagulated blood, the divided vessels were included in fine silk ligatures; "after which," says Dr. Yandell, "the openings in the gut were each closed with the same species of thread, but whether more than one stitch was used, I am not able, at this distance of time, to say." In two weeks the man had so far recovered that it was no longer necessary to visit him.

---

* I am indebted for the above case to my friend and colleague, Professor Yandell. As it occurred more than ten years ago, and no notes were taken of it at the time, it is not so circumstantially reported as could be desired.

The following cases, although they had an unfavorable termination, throw additional light upon this important subject. They occurred in the practice of Sir Astley Cooper, and are recorded in his great work on the Anatomy and Surgical Treatment of Abdominal Hernia, edited by C. Ashton Key, Esq.

Case I.—Strangulated crural hernia in a woman fifty years of age—mortification of the ileum, and excision of the affected part—three interrupted sutures with the ends protruding through the outer orifice—artificial anus—death on the fifth day after the operation.

A woman, fifty years of age, had been laboring under strangulated crural hernia from the first until the eighth of November, when Sir Astley Cooper was requested to visit her for the purpose of performing an operation for her relief. Her features at this time were anxious and collapsed; the pulse was one hundred and thirty a minute; there was great thirst; the abdomen was distended and tender on pressure; the bowels had been obstinately constipated for more than a week; there was frequent vomiting of a yellowish fluid, of fæculent odor; and the tumor was red, hard, and exquisitely painful to the touch. Having laid open the hernial sac, a quantity of liquid fæces immediately escaped from it, which was found to have proceeded from a large circular opening of the ileum, with dark, thickened, and everted edges. After the stricture was fully divided, he cut away the mortified piece of bowel, which was about two inches and a half long, and joined the two fresh ends by three sutures, leaving a small aperture for the evacuation of fæces, and confining the ligature which passed through the back part of the tube next the mesentery to the mouth of the hernial sac. The external wound was closed in the usual manner, except at one point for the passage of alvine matter. She died on the morning of the 12th of November, every thing that she swallowed having in the mean time been speedily discharged at the groin. The integuments over the artificial anus were of a livid color, but not mortified, and she had no passage since the attack. On dissection the protruded part of the tube was found to be firmly glued to the inner side of the sac, and the small bowel above this point highly inflamed throughout. The stomach was pale and contracted; the large intestine was free from disease; and there was no effusion of fluid into the peritoneal cavity, nor any adhesion of the abdominal viscera.

Case II.—Strangulated crural hernia—patient sixty-eight years old—mortification of the bowel—excision of the affected part—three interrupted sutures with the ends protruding through the outer orifice—artificial anus—death in ten weeks.

This case was likewise one of crural hernia; the patient was a female, sixty-eight years of age, and the strangulation had existed for five days. When Sir Astley Cooper saw her, on the 31st of July, she had repeatedly vomited, and there was slight hiccough, with a small and frequent pulse. The tumor was much inflamed, and pitted under pressure. After exposing the bowel, he discovered that it was mortified to the extent of about three-quarters of an inch, and that there were two holes in it, one of which was large enough to admit the blunt end of a probe. Both apertures were of a circular form, and readily permitted the escape of fæcal matter when pressure was applied to the adjoining portions of the tube. With a pair of scissors he cut away the sphacelated piece, and then united the parts by three sutures. The divided edges bled freely, but the hemorrhage was checked when the ligatures were drawn together. The intestine was then pushed as near as possible to the mouth of the hernial sac, and the threads left hanging from the wound. The protruded omentum was cut off, and the external opening every where closed, except at the centre, to allow of the escape of fæcal matter, should it be disposed so to do.

On the second day after the operation a large quantity of liquid fæces passed from the wound, and in a short time afterwards the artificial anus appeared to be fully established the opening into the bowel being large enough to admit the finger. From this time until the twenty-third of September the case presented nothing of any particular interest. At this period the wound was very much contracted, the hole in the bowel was greatly reduced in size, and all discharge of fæces had ceased, owing, as was supposed, to her having eaten some rabbit and roasted apple. She vomited, and the belly became distended. After remaining in this state for forty-eight hours, a large alvine evacuation took place from the wound; but her strength gradually declined, and she expired on the 9th of October. On opening the body, the abdomen was found free from inflammation. The lower part of the ileum formed the artificial anus. The large bowel was much contracted, and contained only a little mucus. The orifices of the intestine were both very small, the lower much more so than the upper.

Who can doubt that the last case would have recovered, if it had been properly managed? Had a few more points of suture been used, the formation of an artificial anus would have been prevented, and nature effected speedy reparation. As it was, the continuity of the tube was interrupted, and when the external opening became greatly reduced in size, as it did a short time before death, obstruction with its whole train of evils was the necessary and inevitable consequence. Even in the first case it is not improbable that recovery might have taken place, if the divided parts had been approximated in such a manner as to prevent the establishment of an artificial anus. During the four days which the patient survived, every thing she drank passed by the preternatural opening, the bowels below remaining in the meantime obstinately constipated. It is true the inflammation might have extended too far before the operation was performed, but this is a mere conjecture, and does not invalidate the belief that, had the wound been carefully sewed up, and the continuity of the canal re-established, restoration might have occurred.

### 3.—*Method of Ramdohr.*

This method derives its name from Ramdohr, an eminent German surgeon, who flourished at the commencement of the last century. It consists in joining together the two ends of the divided bowel by introducing the upper within the lower, and fixing it there by means of a suture, the extremities of which are brought out at the opening in the abdomen. Ramdohr, I believe, was the only surgeon who, until recently, performed this operation on the human subject; his patient, a female, was affected with strangulated crural hernia, and, although he removed two feet of mortified intestine, perfect recovery soon ensued. About a year subsequently to the operation she died of an attack of pleuritis, when the bowel was carefully inspected, and the two ends were found to be beautifully united to each other and to the wall of the abdomen. The preparation was sent to Professor Heister, of the University of Helmstadt, who preserved it in alcohol,* and published an account of it in his Institutes of Surgery. To facilitate the invagination, Ramdohr recommends the division of a small portion of the mesentery along the concave surface of the tube, and the insertion of a piece of candle.

---

*"Et excisa magna intestinorum parte corrupta, binas partes extremas, easdemque sanas, superiori inferiorem insinuata, leniter per injectum filum conjunxit. In abdomen reposuit, filique circumducti ope ad vulnus abdominis attraxit; atque ita non modo efficit ut cum vulnere confervesceret, et ad glutinationem quod mirum videri poterat, intestinum divisum perveniret, sed feminam quaque velut ex ipsis mortis faucibus retraheret, fæcibus postea non per vulnus, sed per anum egredientibus. Mulier illa posta sana vixit; at post annum ex pluritide abiit, atque in inciso cadavere intestina divisa inter se rursus coalita de prehensa sunt: quæ ipsæ mihi una cum parte abdominis cum qua coaluerunt, dono dedit; ea que adhuc in spiritus vini asservo, ut dubitantibus aut discentibus ea semper attendere possim."—Heister, *Institutiones Chirurgicæ*, T. i., p. 768, in 4to.

The objections to this procedure are, first, the impossibility of distinguishing the upper from the lower end; secondly, the difficulty of effecting the invagination; thirdly, the tardy and imperfect adhesion from a serous surface being placed in contact with a mucous; and fourthly, the danger of hemorrhage from the division of the mesenteric arteries. I shall examine these objections in detail.

The difficulty of distinguishing the two ends from each other is always great, if not absolutely impossible. One of the most important signs enumerated by authors is the disproportionate contraction of the inferior extremity. This occurrence, however, although it may occasionally happen, is, nevertheless, exceedingly rare, and cannot therefore be depended upon. I have seldom noticed it in my experiments, and the same remark has been made by others. The contraction is sometimes more conspicuous in the upper than in the lower end, sometimes it is entirely wanting, and in some instances it is nearly equal in both divisions. Professor Bérard of Paris, who was called to a female who had cut out two feet of her small bowels, relying upon the certainty of this sign, was unwittingly led into the error of inserting the inferior into the superior orifice, as was shown by the autopsy, the patient dying in thirty-six hours.*

Louis proposed the administration of a small quantity of olive-oil, to promote the peristaltic action of the alimentary canal. The alvine matter above the seat of the injury would thus be evacuated through the superior orifice, and so lead to its detection. This is, however, to say the least, a tardy and uncertain procedure, and one to which few practitioners of the present day would be likely to trust. Where the stomach is oppressed, as it almost always is in wounds of the bowels, with nausea and vomiting, no medicine, however mild, would be likely to be retained sufficiently long to pass the pyloric orifice. Granting, however, that it might reach the bowel, a number of hours would necessarily elapse before

---

* London Lancet for 1835–'6, p. 45.

it would produce the desired effect. In the meantime the patient would be subjected to the pain and hazard resulting from the exposed condition of the protruded viscus, which should always be returned as speedily as possible; for the longer this is delayed the greater will be the risk of severe peritonitis and the probability that the patient will die from the shock of his wounds. But I need not dwell upon this proposal, as it is altogether unlikely that it will be carried out by any practitioner of the present day.

A recent writer, Mons. Jobert, of Paris, observes* that in a sphacelated hernia the escape of the intestinal contents would show which was the upper end; while, in the case of division by a wound, the method suggested by Louis would be the best, especially if the oil were mixed with some coloring substance, as syrup of violets or orchanet. "In order to distinguish," says Professor Cooper, "the upper end of the intestine from the lower, the proposal is sometimes made to give the patient a little milk, and to observe whether the fluid, after a time, issues from the mouth of the protruded gut."†

Some diversity of sentiment still exists in respect to the absolute necessity of distinguishing the two ends from each other. Jobert thinks it of paramount importance, on the ground that if the inferior be inserted into the superior it will lead to inversion and obliteration of the tube, followed by death from inanition; in proof of which he refers to an experiment by himself on a dog in which this result actually happened. On the other hand, there is now on record at least one example in which the reverse occurred in the human subject. Such, at all events is the probability, for as the patient recovered no decisive examination could of course be made. I allude to the case mentioned by Dr. Pitcher, to which I shall hereafter refer in connexion with Ramdohr's process, and in which the lower portion of the small bowel was inserted into the upper. "I did this," observes Dr.

---

* Op. cit. T. i, p. 85.

† Dict. Surgery, p. 503. New-York edition.

Pitcher," because the lower end had been already, by the butcher's knife, freed from its connexion with the mesentery, in which I found the chief impediment to this mode of junction. The peristaltic contractions occasioned by handling the bowels embarrassed the operation very considerably, but that difficulty was overcome by the manner of passing the ligatures already described."*

In respect to the difficulty of effecting the invagination there is hardly a practical writer that does not fully concur in it. It has been already stated, in a previous section, that when a bowel is completely divided, there is not only retraction of its extremities but also a certain degree of contraction, by which the caliber is sometimes diminished one-half or even two-thirds, or, rather, I might say, almost entirely obliterated. Now any attempt under these circumstances to insert the superior end into the inferior, provided it was always possible, which, as we have just seen, it is not, to distinguish them from each other, would inevitably prove abortive, unless the parts were most forcibly dilated, and even then it would be almost impracticable. Of the truth of this abundant evidence has been furnished me by my own researches, multiplied and repeated as they have been in a great variety of ways and in numerous instances. If any further proof, however, is needed it is only necessary to refer to the experiments of Mœbius, a cotemporary of Ramdohr, and to the more recent ones of Dr. Smith and Mr. Travers, before adverted to, in which they uniformly failed to accomplish this object. "Having divided," says Smith, "the intestine of a dog transversely, I attempted to treat it in the manner spoken of by Mr. Ramdohr, namely, by introducing the upper extremity within the lower; after having procured a piece of candle, as directed by him, it was inserted into that portion of the intestine which was supposed to be the uppermost. I then endeavored to introduce the superior within the inferior, but the extremities of each became so everted that it was utterly impossible to succeed; it was therefore given up, and

---

* American Journal Med. Sciences. vol. x, p. 47.

treated in the way recommended by Mr. John Bell.* To the same import precisely is the testimony of Sir Astley Cooper. "Some years ago," he observes,† "I divided the intestine of a dog, with a view of trying to introduce the one intestine within the other; but I had no sooner made the division than the intestines became everted, and so bulbous at each extremity that I found it impossible to pass one within the other; and that this also takes place in the human subject is proved by a preparation of wounded intestine in the Museum at St. Thomas' Hospital, taken from a man who had been kicked by a horse. The jejunum was ruptured, and it appears everted." Such, indeed, must be the experience of every practitioner who has had an opportunity of witnessing a lesion of this kind, whether in the human subject, or in the lower animals.

To overcome this contraction, Professor Velpeau thinks the best plan would be to seize simultaneously the two principal diameters of the inferior end by their four extremities with an equal number of forceps or hooks. Swelling or distention of the upper end may be prevented by an assistant holding and compressing it, while the operator endeavors to introduce it into its destined situation.‡ This plan, however, is by no means free from objection, since it can only succeed at the expense of much pain to the patient, and the risk of creating unnecessary inflammation. I have tried it in several instances, and this is precisely the conclusion at which I have arrived.

The third objection to this proposal is the apposition of a serous with a mucous surface. This constitutes no little impediment to the reparative process, which can be accomplished only after a long time, and then probably in a very imperfect manner. Indeed, Mr. Lawrence and others are inclined to suppose that direct union cannot be effected at all under these circumstances, asserting that the success depends

---

* Inaugural Essay, Caldwell's Collection, p. 296.

† Treatise on Hernia, p. 54. London, 1827.

‡ Medecine Operatoire, T. iv, p. 134.

altogether upon the extent and firmness of the collateral adhesions; an opinion which, there is reason to believe, is in the main well founded.

Lastly, the upper part which is to be inserted into the lower, must be separated from the mesentery, a procedure which sometimes exposes the patient to considerable risk from hemorrhage. Of this fact my own experiments have afforded me a number of striking and convincing proofs. Baron Boyer of Paris, in attempting to put this method into execution, tied not less than seven or eight arteries, and yet his patient died from effusion of blood into the abdomen.

Velpeau states that he saw this method tried at the St. Louis Hospital of Paris, by Professor Richerand, upon a patient who died the following day.* Baron Boyer executed it with no better success.† His patient, an athletic brasier, about forty-five years of age, had been affected with strangulated inguinal hernia for three days, and on exposing the bowel he found that it was mortified. He accordingly made an incision to the extent of four inches into the sphacelated part, and thus allowed the escape of its contents, to the great relief of the individual. The operation over, he administered mild opening medicines, both to evacuate the alimentary canal, and to enable him to distinguish the upper extremity of the gut, which, however, was already sufficiently obvious from its dilated condition. The next day he cut away the mortified portion, and united the two ends according to Ramdohr's method, introducing the superior, supported by a cylinder of card, into the inferior. The operation, however, was not only tedious but extremely painful, and when completed, he found it impossible to return the gut, distended as it was by the foreign body, without a considerable enlargement of the ring. The patient grew decidedly worse during this second operation; the symptoms of strangulation, which had been relieved by the free discharge of fæcal matter through the

---

* Op. cit. T. iv, p. 134.

† Traité des Maladies Chirurgicales, T. viii, p. 134.—Lawrence on Ruptures, p. 359.

mortified part, were soon renewed, and destroyed the patient in sixteen hours. The dissection revealed inflammation of the intestines and a slight effusion of blood into the peritoneal cavity.

Flajani, of Rome,* also tried the artifice on several occasions, but death was invariably the consequence. He experienced great difficulty in his attempts to invaginate the divided extremities of the bowel, and speaks of the practice in terms of decided condemnation.

Notwithstanding these difficulties and disasters, it would seem, from the testimony of a recent writer, Professor Velpeau, that Laviellé, Chemery-Havé, and Schmidt have each reported a successful example in support of the practice. Another, which occurred in our own country, was published a few years ago, by Dr. Zina Pitcher of the United States army, and, from the manner in which it was treated, reflects much credit upon that gentleman. The following is an abstract of it.†

Nicholas Miller, a citizen of the Cherokee nation, was stabbed on the 22d of June, 1831, with a butcher's knife, which, entering the abdomen at the left internal ring, passed upwards and inwards towards the median line, making a wound three inches in length in the skin, and another still more extensive in the peritoneal sac, followed immediately by a protrusion of several feet of intestines. The knife had divided the ileum diagonally, and separated two inches of the lower portion of the mesentery. The fold of intestine in contact with this was cut on its convex side two-thirds across; two other convolutions were transpierced, and the descending colon was partially opened in the direction of its circular fibres. Three branches of the mesenteric artery bled pretty profusely, and were included in separate ligatures, the ends of which were cut off close to the knots. The extremities of the ileum were brought together by passing a needle, armed

* Collezione d'Osservazioni, &c., di Chiurgia, T. iii, p. 60. Roma, 1802.

† American Journal of the Medical Sciences, vol. x, p. 42.

with a thread, through the upper portion from without inwards, thence into the lower part and out again, including half an inch of intestine in the stich, after which it was returned through the upper end from within outwards. Three sutures of this kind made the intus-susception complete. The extremities of the ligatures were cut off near the peritoneal surface. The other openings of the small bowel were closed with the continued suture, the ends of which were left long, and so tied as to hang within the tube. The wound in the colon was united with a single stich. The prolapsed intestines were next sponged with warm milk and water and returned into the abdomen; a few pieces of the omentum which had been injured by the knife were excised, and the edges of the outer wound approximated by half-a-dozen turns of the continued suture. The external ligatures were detached on the fifth of July, and by the eleventh of the month the wound in the abdomen was completely healed.

Laviellé's case occurred at Mainbaste, in the department of Landes, in France, and is recorded in the forty-third volume of the "Journal Géneral de Médecine." The following notice of it is taken from Jobert's treatise on the surgical diseases of the alimentary canal.* The patient was affected with inguinal hernia of the left side, which at length became strangulated. The tumor was of considerable volume; gangrene supervened, eventuating in the sloughing of the common integuments, and the effusion of fæcal matter. A longitudinal incision being made down upon the parts, a coil of intestine, a foot long and completely sphacelated, was removed with the scissors, when the extremities of the tube were fastened to the outer opening with a thread carried through a fold of the mesentery. At the expiration of twenty-four hours, Laviellé invaginated them by inserting the superior within the inferior, and keeping them in contact with the ligature previously attached to the mesentery, the ends of which, after the replacement of the gut, were brought out at the wound

* T. i, p. 85.

in the abdomen. The next day the man walked to the guard-house, and continued so to do regularly during the treatment. The cure was completed in sixty days.

### 4.—*Method of Le Dran.*

Le Dran was a warm advocate of what is denominated the *looped suture*, of which he was the inventor.* Whether he ever employed it in the human subject for purposes of this kind, I am unable to say, as I have not before me a copy of his works. It is not improbable, however, that he did. To perform this suture, as many needles should be used as it is intended there should be stitches; they should be round, straight, and slender, and furnished each with an unwaxed thread a foot long. The lips of the wound being held by the surgeon and his assistant, as many ligatures are passed through them as may be considered requisite, with the precaution to let the intervals between each two of them not exceed a quarter of an inch. When the sutures are all introduced and the needles removed, all the threads of one side of the cut are tied together at their ends, and then those of the opposite side, after which the whole are united and twisted into a sort of cord. The stitches by this procedure are approximated to each other, and the divided extremities of the intestine thrown into plaits, by which the edges of the solution of continuity are, it is said, prevented from gaping. The bowel being replaced, the threads are secured to the bandage

* Traité des Operations Chirurgicales. Paris, 1742.

which is put over the dressing, and the outer wound is closed in the ordinary manner. When the injury is sufficiently repaired, which rarely happens under five or six days, the ligatures are untwisted, and all the ends on one side cut off on a level with the skin, after which the others are to be slowly and cautiously withdrawn.

The advantages which Le Dran claims for this suture are the two following; first, that the twisting of the threads, as stated above, produces a slight puckering of the surface of the injured bowel, by which the re-union of the edges of the wound is more effectually and speedily secured; and secondly, that the ligatures may be withdrawn with so much facility as not to interfere, in the slightest degree, with the adhesive process. These advantages are, it is obvious, altogether chimerical, for this puckering of the bowel, instead of promoting the apposition of the edges of the wound, as is contended by Le Dran, has the effect of separating them from each other, and thereby increasing the danger of fæcal effusion. The removal of the ligatures, notwithstanding the ease with which it is accomplished, must also have a tendency to break up the tender adhesions of the part, if not to excite undue irritation in the peritoneum. Besides these objections, which are in themselves sufficiently serious to prevent any future recourse to this method of treatment, it is alleged that it is almost always followed by such a diminution of the caliber of the alimentary canal as to interfere essentially with the passage of its contents. Mons. Velpeau, in his Surgical Atlas, has delineated this suture, but whether with the design that it should be adopted in practice, or as a piece merely of scientific curiosity, I am unable to say. He has not made any special mention of it in connexion with the subject in his "Médicine Opératoire."

5.—*Method of Bertrandi, or "La Suture à Points Passés."*

Another method, which appears to have been a good deal employed at one time, was devised by Bertrandi, and is usually described by the French writers under the phrase of "la suture à points passés." It differs from the continued suture merely in having all the loops laterally, and in drawing together only the internal lips of the wound, the outer remaining apart; or, in other and more simple language, the ligature is passed through, not over the margins of the solution of continuity, as in the common operation. The method of Bertrandi has been advocated by Sabatier, Desault, Boyer, and several other surgeons, though they have not, I believe, adduced any facts in illustration of its efficacy. Boyer says* that it possesses the advantage of keeping the edges of the wound together and of promoting their adhesion with the surrounding parts, at the same time that it does not occasion any puckering of the bowel, or diminution of its caliber; and for these reasons he seems to be inclined to give it a preference over other procedures.

The "suture à points passés" is performed with a round, straight needle, armed with a waxed thread. As a preliminary step, the surgeon adjusts the edges of the wound, placing them parallel and in close contact with each other. For this purpose he takes hold of one extremity of it himself, and intrusts the other to an intelligent assistant. The needle is then carried somewhat obliquely across the lips of the opening, about the fifth of an inch from its extremity: having done this it is brought back in the same manner, and thus it is passed alternately from one side to the other until the whole track is closed up, the operation being similar to that employed by a tailor in sewing together two pieces of cloth. The interval between the respective stitches should not exceed two lines, or the sixth of an inch, otherwise fæcal matter may escape into the abdomen. The intestine being replaced, the extremities of the suture are brought out at the external opening, where they are secured by a strip of adhesive plaster. In a few days one of them should be cut off close to the wound, and the other gently

* Maladies Chirurgicales, T. 7, p. 379.

pulled to promote its separation. Some of the successors of Bertrandi recommend that the ligature should be passed through the edges of the outer orifice, to prevent the bowel from slipping out of the reach of the surgeon; a precaution which can only be necessary when the patient is very young or restless.

To obviate the danger of destroying the feeble and imperfect adhesions of the intestine, incurred in withdrawing the suture in the manner suggested by Bertrandi, it occurred to Béclard that it might be advantageous to use two ligatures, one white, the other colored. The mode of performing the operation does not differ in other respects from that which we have just described. When the time for removing the threads has arrived, the surgeon withdraws them in opposite directions, taking hold of the white one, for example, with the left hand, and of the colored with the right. The result of this traction is that the adhesive process is scarcely at all disturbed, while the reverse must always happen when the suture is detached in the manner recommended by Bertrandi.

With the exception of Boyer, I do not know that the method of Bertrandi has any advocate at the present day. I have not tried it upon any of the inferior animals, and we are not in possession of any facts which warrant its employment in the human subject.

### 6.—*Method of the Four Masters.*

The method of the four masters—"*Quatre Maitres*"—as it is termed, which is usually attributed to Duverger, who was the first to revive it after it had fallen into neglect, consists in stitching the divided ends of the bowel over a piece of trachea, either of the calf or of some other animal. What the precise length of the tube was I am unable to say, but in all probability it did not exceed two inches. In its diameter it was a little smaller than the alimentary canal, into which it was intended to be introduced, and previously to using it it was well dried and varnished, to prevent it from too readily imbibing moisture. Three ligatures were passed through it equidistant from each other, and armed each with a small curved needle. The piece of trachea thus prepared was inserted into the ends of the bowel, where it was secured by three interrupted sutures made by passing the needles from within outwards, about three lines from the edges of the wound, which were held together by an assistant. The ends of the threads were cut off close to the knots, and the parts reduced by pushing the lower end in first.

This method of the four masters is said to have been successfully employed by Duverger in a case of strangulated hernia, in which a part of the bowel was affected with gangrene.* In the account of this process, as given by Dupuytren, in his Treatise on Gun-shot Wounds,† the inferior extremity is directed to be drawn nearly half an inch over the superior, placing thus, as in the operation of Ramdohr, a mucous surface in contact with a serous. The surgeon, also, is made to use a single suture, instead of three, as stated above, and the upper end of the gut is to be carefully distinguished from the lower.

This method, slightly modified, was successfully employed by Sir Astley Cooper upon a dog. He used a cylinder of isinglass instead of a calf's trachea, upon which he made three sutures, one at the mesentery, and another at each side of the bowel, which was then returned into its natural situation. In three days the animal took food, had regular stools, and on the sixteenth day he was killed, when the united parts were shown by Sir Astley to his students. No advantage appeared to result from the cylinder of isinglass, as it became shut by the contraction of the intestine, and the experiment was therefore never repeated.‡

---

* Dict. de Med. et de Chir. Pratiq., T. 13, p. 267.

† T. i., p. 194.

‡ On Hernia, p. 51.

Sabatier proposed, as a substitute for the piece of trachea, recommended by the four masters, a roll of paste-board, which he advised to be well varnished with oil of turpentine, or some other tenacious fluid, and fastened to the bowel with a single stitch. Watson, an English writer, speaks favorably of a canula of isinglass. Some of the older surgeons were in the habit of employing a tube of elder-wood; others a piece of tallow candle. Rogers, Garnier, and Théodore recommend the use of the elder-wood to defend the suture from the injurious effects of the fæcal matter, of which they appeared to have much dread.

The method of the four masters, somewhat modified, is warmly advocated by Chopart and Desault, in their Treatise on Surgery. The improvement which these two distinguished men suggested, but which was never, I believe, carried into effect by them upon the human subject, consists in passing two fine needles, armed with a silk ligature, twelve inches long, through the centre of the paste-board cylinder, and bringing them out respectively three lines above and below their place of entrance. The ligature will thus be found to be attached to the artificial tube, without crossing its cavity, or interfering in any wise with the transmission of fæcal matter. The two needles are next carried through the upper part of the bowel, equidistant from each other, and at a point from the wound equal to the half of the length of the cylinder. The latter is now to be engaged in the upper portion of the intestine, after which the lower end is to be pierced in the same manner, but a little farther from the seat of the injury, and the remainder of the tube to be introduced along with five or six lines of the inferior extremity of the gut. Should this invagination be attended with much difficulty, on account of the mesentery, the latter should be detached to the requisite extent, and the operation finished in the manner already stated, care being taken to tie any obstinately bleeding vessels. The parts, when returned, should be kept in exact apposition with the external wound, to promote their adhesion, an object which may be readily accomplished by securing the ends of the ligature to the surface of the abdomen.*

### 7.—*Method of Palfin, Bell, and Scarpa.*

J. Palfin, author of the "Anatomie Chirurgicale," thought it of much less importance to sew up the wounded intestine than to stitch it to the wall of the abdomen. In conformity with this belief he advises the surgeon to carry a waxed thread, armed with a needle, through the edges of the solution of continuity at their centre, and after tying it into a simple slip-knot, to bring the ends out at the external opening, where they are to be secured by an adhesive strip. He entertained the singular notion that the divided ends never united with each other, but that the cure was effected solely by the adhesions which they formed to the surrounding parts. This plan, which certainly possesses the merit of great simplicity, he considered as equally applicable to transverse and longitudinal wounds.†

This method of the old French surgeon found a warm advocate in that great luminary of the profession, the late Mr. John Bell, who, however, does not appear to have been aware that it had been previously recommended; at all events, he has not any where alluded to Palfin or his writings in connexion with the subject. Like his Gallic predecessor, he suggests that only a single stitch should be taken, and that the thread should be brought out at the external opening; adding, in his own expressive language, that if there be in all surgery a work of supererogation it is this operation of sewing up a wounded gut.‡ This plan he advises not only where there is a simple slit-like aperture in the bowel, the

---

* Tavernier's Operative Surgery, translated by the author, p. 276. Phila. 1829.

† Anatomie Chirurgicale, T. ii, p. 76. Paris, 1743.

‡ Discourses on the Nature and Cure of Wounds, vol. ii, p. 80. Walpole, N. H., 1807.

kind of injury most commonly met with, but where it is divided in its entire cylinder. He is of opinion that it is only necessary to keep the wound of the intestine neatly and closely in contact with that in the wall of the abdomen, when the parts will gradually adhere, affording at the same time an opportunity for the escape of fæcal matter. He contends that sewing up the breach in the intestine firmly with a needle and thread is absurd, and that the mere pressure upon the abdominal viscera will keep the edges of the wound so close to the peritoneum as to insure their re-union. But is this the practice generally pursued by surgeons, or, rather, is it not universally abandoned, for the sufficient reason of its entire inadequacy? If there ever was an error committed by any writer more serious, culpable, and mischievous than another, it is most assuredly this of Mr. John Bell, who while criticizing and condemning, in no measured terms, the advice and practice of others, has himself fallen into a most strange delusion. Had he performed the operation in a single instance upon the human subject, or upon an inferior animal—an experiment from which he affects so much to shrink—he would have become fully sensible of its danger and insufficiency. That the operation, as recommended by this eminent surgeon, might occasionally be attended with success is not improbable, but that it should not be trusted to in the present enlightened state of the healing art must be obvious to all who will be at the trouble to investigate it. Independently of the great risk of fæcal effusion into the peritoneal cavity, there are few cases, if any, in which it would not be followed by an artificial anus, an occurrence which need never attend enteroraphy when performed in the manner previously pointed out.

Although both Smith and Travers had already exposed the insufficiency of this mode of procedure, I was determined, if possible, to throw additional light upon it, and with this view instituted several experiments, the results of which, as will be here seen, fully confirm those of the above investigators.

Experiment I.—Having obtained a small slut, a fold of the ileum was drawn out of the abdomen, and divided through its entire cylinder. A single stitch was then carried through the everted edges, at the point opposite to their attachment to the mesentery, when the ends of the ligature were tied and left protruding at the external orifice, which was secured in the usual manner. In thirty-five hours the animal expired, having in the meanwhile suffered severe pain and refused such food as was offered her. The opening of the abdomen was followed by the escape of a considerable quantity of fœtid gas; and the peritoneal sac, which exhibited marks of high inflammation, contained more than an ounce of fluid and solid fæces. The edges of the wound were red, besmeared with thick and ropy mucus, and at least three lines apart at the widest portion of the breach. No attempt at reparation was visible.

Experiment II.—The above experiment was repeated upon a small but full-grown dog, which died in twenty-four hours after the operation. A considerable quantity of thin alvine matter was found in the abdomen, as in the preceding case, the peritoneum was extensively inflamed, several coils of intestine adhered slighly to each other, and the lips of the wound were deeply injected, with marked eversion of the mucous membrane, but no incrustation of plastic lymph. It is worthy of remark that, neither in this nor in the preceding experiment, was there any discharge of fæces through the external wound.

Experiment III.—A young dog of moderate size was submitted to the same experiment as the two preceding, with this difference, that the incision passed only through two-thirds of the intestine, producing a wound about an inch and three-eights in extent. For the first twenty-four hours he was apparently well, being lively and cheerful, but afterwards symptoms of indisposition came on, and he died early on the third day. The lips of the wound, red and injected, were separated fully a line and a half at their middle; semi-fluid fæces with some water which the animal had drank, had escaped into the abdomen, and the peritoneum, especially in the vicinity of the injury, displayed strong marks of inflammation. The external wound had a red angry appearance, from the passage, no doubt, of fæculent matter, which was discharged through it for ten or fifteen hours before death.

In an experiment of this kind performed by Mr. Travers,* the animal survived only a few hours. The peritoneum appeared highly inflamed, adhesions existed among the neighboring folds, and lymph was deposited in masses upon the sides of the injured gut. A quantity of bilious fluid was found among the abdominal viscera together with some other extraneous substances, and a worm was depending from one of the apertures of the gut, which had receded to the utmost and were of a circular form.

In three experiments by Dr. Smith† instituted with a view of ascertaining the merits of Mr. Bell's treatment, one of the dogs died at the end of the first day, the other on the fifth day, and the third on the seventh day. In all, the intestines were very much inflamed, from the effusion of fæcal matter into the peritoneal sac. In one of the animals, that namely which lived longest, one part of the injured intestine had contracted adhesions to the external wound, allowing thereby a slight discharge of fæces in that direction.

Thus, in seven experiments, all conducted, there is reason to believe, with the requisite care and skill, not a single one had a favorable termination. Nor is this surprising when we consider the circumstances which invariable attend lesions of this description; we have already seen that punctures of the bowel, more than a third of an inch in length, are almost constantly followed by fæcal extravasation, and the same thing it will be recollected is apt to happen in more extensive wounds treated with the interrupted suture, when the interval between each two respective ligatures exceeds three or four lines. That Mr. Bell should have committed

---

* Op. cit., p. 116.

† Caldwell's Medical Theses, p. 296.

such an error is not to be wondered at when we remember the period at which he wrote; he instituted no experiments on any of the inferior animals to elucidate the subject, and the beautiful researches of Travers, Thomson and Smith had either not been made, or no notice of them had appeared. He sinned, therefore, because he had not the requisite light to guide him. But it is otherwise with Professor Cooper, of London.* In sanctioning, as he does, the practice of John Bell, he is instrumental in perpetuating an error for which modern surgery can find no excuse, and which deserves to be reprobated in the strongest terms, from the pernicious tendency which it must exert upon the younger members of the profession when inculcated by an authority so respectable and influential.

Professor Gibson, of Philadelphia, appears to be disposed to advocate the same kind of practice.† "Should a case present itself" says he, "which, from the extent of the wound and other circumstances, seemed to require a suture, I should be inclined to follow the plan of Mr. Bell, and simply employ one or more tacks of the *interrupted* suture, merely for the purpose of connecting the wound in the gut slightly to the external wound."

Still more extraordinary and unaccountable is the plan of procedure proposed by Professor Scarpa, of Italy.‡ This celebrated surgeon has offered a variety of arguments against sewing up the wound at all, and asserts that in all cases of injury of the intestinal canal, whether the opening be longitudinal or transverse, a suture is always not only useless, but even dangerous and fatal. Great evils, he thinks, arise from the passage of the ligatures, however few, across the delicate and sensitive tunics of the bowels, which are thus exceedingly apt to become inflamed, and to propagate the morbid action rapidly to the surrounding viscera. He affirms that the expe-

* First Lines of Surgery, vol. ii, p. 74.

† Institutes of Surgery, vol. i, p. 119. 1838.

‡ Treatise on Hernia, translated by Wishart.—Cooper's First Lines of Surgery, vol. ii, p. 71.

rience of several ages clearly proves that nearly all who have been subjected to enteroraphy have died in the severest agony, and that the few who have recovered have escaped, not in consequence of the operation, but in despite of it. I do not deem it necessary to enter into any formal refutation of these singular views of the illustrious professor of Pavia. I must only express my surprise that they should have been entertained and promulgated after the publication of the researches of Mr. Travers, which shed so much light upon the subject, and so emphatically inculcate the indispensable importance of the suture in all wounds of the intestines, even when of comparatively small extent. It is one of those remarkable circumstances which not unfrequently occur in our profession, and which can only be explained by a reference to the infirmities and prejudices of our nature. The case of Scarpa is on a par precisely with that of John Bell. When this eminent surgeon was at such pains to criticize and condemn the practice of his name-sake, Benjamin Bell, of Edinburgh, in regard to the present topic, he had probably little idea that the verdict of the profession would, in less than a quarter of a century, entirely reverse his decision, and treat him as unsparingly as he did his Scotch cotemporary.

(*To be continued.*)

## Figures and Tables

**Figure f1:**
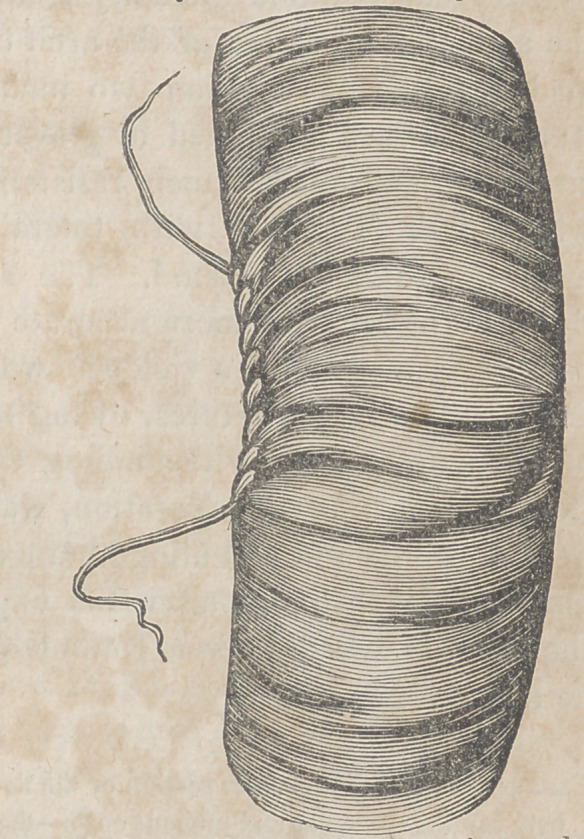


**Figure f2:**